# Diabetic Foot Ulcers Among Patients With Diabetes Mellitus Attending Primary Healthcare Centers in Bahrain: A Cross-Sectional Study

**DOI:** 10.7759/cureus.105815

**Published:** 2026-03-25

**Authors:** Mahmood Alawainati, Lamees Almuqahwi, Lujayn F Juma, Sarah Obaid, Marya Radhi, Lenah Mohammed

**Affiliations:** 1 Medicine, Royal College of Surgeons in Ireland, Manama, BHR; 2 Family Medicine, Primary Healthcare Centers, Manama, BHR; 3 Nursing, Primary Healthcare Centers, Manama, BHR; 4 Internal Medicine/Respiratory Unit, Salmaniya Medical Complex, Manama, BHR; 5 Medicine, Mansoura University, Mansoura, EGY; 6 Internal Medicine, Arabian Gulf University, Manama, BHR

**Keywords:** amputation, diabetes mellitus, diabetic foot, noncommunicable diseases, skin abnormalities

## Abstract

Background: Diabetic foot ulcer (DFU) is a serious complication of diabetes that is associated with an increased risk of amputation and morbidity. Early identification and management are essential to prevent DFU and its complications. This study aimed to determine the prevalence and associated factors of DFU among diabetic patients attending primary healthcare centers (PHC) in Bahrain.

Materials and methods: A cross-sectional study was conducted across all PHCs in Bahrain between July and September 2025 using a cluster sampling technique. Adults with diabetes attending diabetes clinics were included. Data on demographics, comorbidities, clinical examination, laboratory values, and treatment were collected using a standardized form. Wagner's classification was used to grade the severity of DFUs after clinical assessment.

Results: A total of 601 patients were included, with a median age of 61 years; most were males (56.6%), Bahraini (85.4%), and had type 2 diabetes (96.7%). Dyslipidemia (77.5%), hypertension (66.7%), and ischemic heart disease (10.1%) were the most common comorbidities. DFU was noted in 5.3% (n=32) of the participants; 15 had superficial ulcers, 10 had deep ulcers, and seven had amputated limbs/toes. In multivariable logistic regression, insulin use (OR 2.555, p = 0.042), ischemic heart disease (OR 3.752, p = 0.016), and skin changes (OR 8.166, p < 0.001) were independently associated with DFUs.

Conclusion: DFU is relatively uncommon among diabetic patients attending PHCs in Bahrain. Ischemic heart disease, foot skin changes, insulin use, and low hemoglobin were associated factors of DFU risk. Routine screening for these high-risk clinical features should be performed by physicians and nurses caring for patients with diabetes to reduce morbidity and prevent severe outcomes.

## Introduction

Diabetes mellitus (DM) is a chronic metabolic disease that is characterized by hyperglycemia due to impaired insulin secretion, insulin resistance, or both [1]. It is linked to several complications, including cardiovascular, neurological, immunological, and metabolic complications [1]. A diabetic foot ulcer, defined as a partial- or full-thickness wound of the foot in a person with diabetes, is a common diabetes-related complication affecting more than 18 million people globally [2].

The pathogenesis of diabetic foot is multifactorial, involving vascular insufficiency, peripheral neuropathy, and impaired immunity. It begins when minor foot injuries go unnoticed due to underlying neuropathy. Because of impaired vascular function and reduced immunity, wound healing is delayed, and ulcers may develop. If left untreated, secondary infection and gangrene might occur [2-4].

Many epidemiological studies assessed the prevalence of diabetic foot ulcers among patients with DM. A systematic review of 36 studies that included more than 11,000 patients from 23 countries reported a pooled prevalence of diabetic feet at risk of ulcer of more than 53%, with the lowest rates in Africa and the highest in America [5]. Another systematic review of 67 studies and approximately 1 million patients from 33 countries reported a prevalence of 6.3%, with higher rates in males and in patients with type 2 DM [6]. A cross-sectional study conducted in Kuwait, Qatar, and Saudi Arabia found that 2.9% of patients had diabetic foot ulcers and 43% had diabetic neuropathy [7]. In Turkey, a higher prevalence of diabetic foot ulcers was reported (>17%) [8].

Some studies assessed the risk factors of diabetic foot ulcers. The literature found that patients with foot ulcers had a longer DM history, were older, and had a lower body mass index compared to their counterparts. Additionally, diabetic foot ulcers were common among hypertensive patients, smokers, and patients with chronic kidney disease [6]. The presence of tinea pedis, a previous history of foot ulcers, and the presence of peripheral vascular disease were also predictors of foot ulcers in patients with DM [8].

Although diabetic foot ulcers are the most common cause of non-traumatic lower limb amputations and are associated with a lower quality of life, such complications are preventable with early diagnosis and treatment [9]. This is why the American Diabetes Association (ADA) guidelines emphasize annual comprehensive foot examinations, including skin inspection and neurological and vascular assessments, to identify risk factors for ulcers and amputations and reduce the incidence of diabetic foot ulcers [10].

Despite being relatively common among patients with diabetes and being associated with significant morbidity, prolonged hospitalization, and increased healthcare costs, few studies were conducted to determine their prevalence, risk factors, and predictors among diabetic patients in Bahrain. Therefore, this study aimed to determine the prevalence and predictors of diabetic foot ulcers among patients with DM attending primary healthcare centers (PHCs) in Bahrain.

## Materials and methods

A cross-sectional study was conducted in all PHCs between July and September 2025. In Bahrain, there are 26 health centers distributed across four governorates. In each health center, non-communicable disease (NCD) clinics manage patients with diabetes and are run by specialized diabetes nurses and family physicians.

Adult patients aged 18 years and older who attended NCD clinics during the study period were included. The study months were selected randomly from the calendar year. The chosen period was selected randomly. Pregnant patients and those with any emergency conditions were excluded.

DM was diagnosed according to ADA criteria, and the duration of diabetes was calculated from the diagnosis to the data collection period. Wagner’s classification was used to diagnose and grade diabetic foot. In Wagner’s classification, grade 0 indicates no ulcer, although cellulitis or deformity may be present. Grade 1 indicates a superficial ulcer, grades 2 and 3 indicate deeper ulcers, and grades 4 and 5 indicate gangrene. The diabetic foot ulcers were diagnosed based on clinical assessment.

Assuming a 95% confidence level, a 5% margin of error, and an estimated prevalence of 50%, the calculated minimum sample size was 385 participants [5,7]. To increase the power, a sample size of 500 was targeted.

All 26 PHCs were included, and two months within the study year were randomly selected as time clusters for participant recruitment.

Specialized diabetic nurses examined all patients for the presence of diabetic foot, and family medicine specialists assessed cases of diabetic foot ulcers for further management. Neurological and vascular examinations, including the monofilament test and Doppler exam, were done by specialized diabetes nurses. Retinopathy was assessed by specialized ophthalmology nurses. Renal complications were assessed by laboratory tests. Skin changes, including dryness, fissures, diabetic dermopathy, and necrobiosis lipoidica diabeticorum, were assessed clinically by physicians. Physicians assessed neuropathic symptoms such as numbness, tingling, and weakness, as well as risk factors such as smoking and alcohol consumption, through direct patient interviews, while medication data were retrieved from the medical records.

A pilot study was conducted on 10 patients to determine the feasibility of the data collection process. The sequence of questions in the data collection tool was modified accordingly. The 10 participants were excluded from the final analysis.

Data analysis was conducted using IBM SPSS Statistics for Windows, Version 26 (Released 2019; IBM Corp., Armonk, New York). Data were entered into SPSS, coded, cleaned, and tested for normality. Categorical variables were presented as frequencies and percentages, while continuous variables were expressed as medians with interquartile range (IQR). Shapiro-Wilk and Kolmogorov-Smirnov tests were used to assess the normality of the data distribution. As appropriate, the chi-squared test, Fisher’s exact test, or Mann-Whitney test was used to determine statistical significance. Then, logistic regression analysis was performed to identify predictors of diabetic foot. All significant univariate variables were included in the regression analysis. The final multivariable logistic regression model obtained after variable selection was reported. Multicollinearity among the independent variables was assessed using the variance inflation factor (VIF) and tolerance. The VIF values ranged from 1.05 for smoking to 1.37 for the monofilament test, and the tolerance values ranged from 0.73 for the monofilament test to 0.95 for smoking. All values are well within acceptable limits, indicating no significant multicollinearity among the predictors in the model. For all statistical tests, a P-value of <0.05 was considered statistically significant.

## Results

A total of 601 patients were included, with a median age of 61 years. Most patients were male (340, 56.6%), Bahraini (513, 85.4%), and had type 2 DM (581, 96.7%); the median duration of diabetes was 11 years. Regarding lifestyle factors, 84 (14%) of the patients were current smokers, and 13 (2.2%) patients reported alcohol consumption. Additionally, dyslipidemia (466, 77.5%), hypertension (401, 66.7%), and ischemic heart disease (61, 10.1%) were the most common comorbid conditions among the participants. Metformin (466, 77.5%), sulphonylureas (259, 43.1%), and DPP-4 inhibitors (256, 42.6%) were the most commonly prescribed medications (Table 1).

**Table 1:** Baseline Characteristics of the Participants With Diabetes Mellitus in Primary Care in Bahrain ACEI: angiotensin-converting enzyme inhibitors; ARBs: angiotensin receptor blockers; DPP-4: dipeptidyl peptidase 4; SGLT-2: sodium-glucose cotransporter 2; GLP-1: glucagon-like peptide-1

Variables		TN=601, n (%)
Age, median (IQR)		61 (54–68)
Sex	Male	340 (56.6)
	Female	261 (43.4)
Nationality	Bahraini	513 (85.4)
	Non-Bahraini	88 (14.6)
Smoking	Yes	84 (14)
	No	468 (77.9)
	Ex-smoker	49 (8.2)
Alcohol	Yes	13 (2.2)
	No	581 (96.7)
	Ex-drinker	7 (1.2)
Type of diabetes	1	20 (3.3)
	2	581 (96.7)
Duration of diabetes in years, median (IQR)		11 (7–20)
Diabetes medications	Metformin	466 (77.5)
	Sulfonylurea	259 (43.1)
	DPP-4 inhibitors	256 (42.6)
	Insulin	225 (37.4)
	SGLT-2 inhibitors	54 (9)
	GLP-1 agonists	31 (5.2)
Other medications	Statins	481 (80)
	ACEI/ARBs	264 (43.9)
	Aspirin	184 (30.6)
	Clopidogrel	7 (1.2)
	Calcium Channel Blockers	125 (20.8)
	Diuretics	75 (12.5)
	Beta blockers	44 (7.3)
Comorbid conditions	Hypertension	401 (66.7)
	Dyslipidemia	466 (77.5)
	Chronic Kidney Disease	22 (3.7)
	Ischemic Heart Disease	61 (10.1)

As shown in Table 2, approximately one in four patients (145, 24.1%) reported sensory symptoms, including burning, numbness, or tingling, while 36 (6.0%) had intermittent claudication and 9 (1.5%) experienced rest pain. Skin abnormalities were noted in 102 (17%) participants, infections in nearly 41 (6.8%) of the patients, and gangrene in 7 (1.2%) patients. Approximately 32 (5.3%) of the patients had diabetic foot ulcers. Most patients had normal monofilament test findings (573, 95.3%), normal retinal examinations (512, 85.2%), and normal Doppler results (586, 97.5%).

**Table 2:** Diabetic Foot Symptoms, Signs, and Examination Findings Among Patients With Diabetes in Primary Care in Bahrain * Burning, numbness, and tingling

Variable		TN=601, n (%)
Foot symptoms	Sensory symptoms*	145 (24.1)
	Intermittent claudication	36 (6)
	Rest pain	9 (1.5)
Foot signs	Skin Abnormalities	102 (17)
	Infections	41 (6.8)
	Foot ulcers	32 (5.3)
	Gangrene	7 (1.2)
	Foot deformities	5 (0.8)
Monofilament Test	Normal	573 (95.3)
	Abnormal	28 (4.7)
Eye Exam—Retina screening	Normal	512 (85.2)
	Abnormal	89 (14.8)
Doppler ultrasound of the dorsalis pedis artery	Normal	586 (97.5)
	Abnormal	15 (2.5)

**Figure 1:**
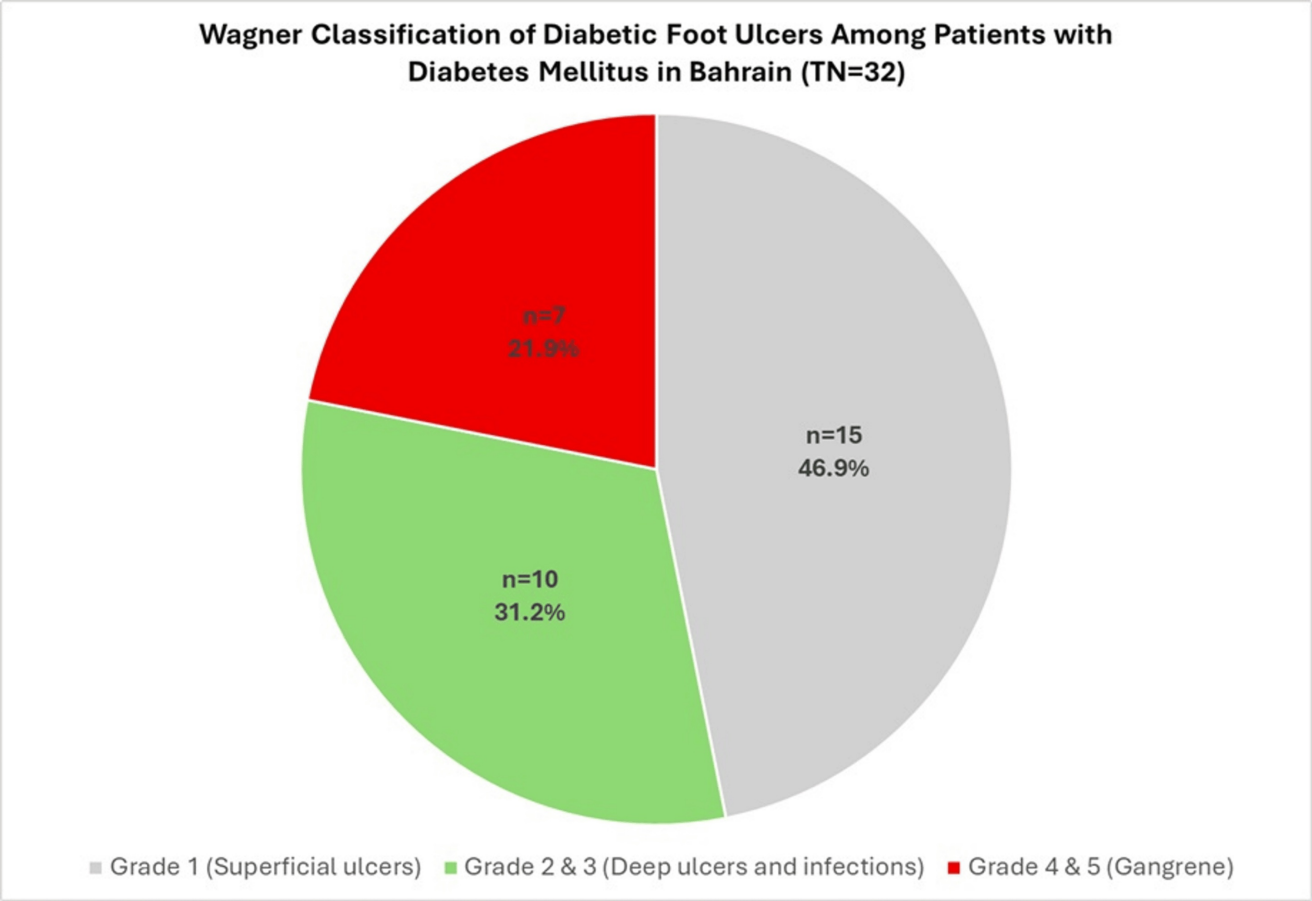
Wagner Classification of Diabetic Foot Ulcers Among Patients With Diabetes Mellitus in Primary Care in Bahrain

As shown in Figure 1, nearly half of the patients with diabetic foot ulcers (15, 46.9%) had grade 1 ulcers, while 10 patients (31.2%) had grade 2 or 3 ulcers. Grade 4 and 5 ulcers were noted in 7 (21.9%) patients.

Univariate analysis showed that smokers (P=0.021), metformin users (P<0.001), and patients taking insulin (P=0.003) had a higher prevalence of diabetic foot ulcers. Moreover, aspirin use was also higher among patients with ulcers (P=0.001). Patients with ischemic heart disease had a significantly higher prevalence of foot ulcers than those without ischemic heart disease (p=0.004). Patients with abnormal monofilament test results (P<0.001) and abnormal Doppler findings (P=0.010) were associated with higher rates of foot ulcers. Additionally, patients with skin abnormalities had higher rates of diabetic foot ulcers (p<0.001) (Table 3).

**Table 3:** Association Between Baseline, Clinical Characteristics, and Presence of Foot Ulcer * Burning, numbness, tingling ** Mann-Whitney test *** Chi-square test **** Fisher's exact test ACEI: angiotensin-converting enzyme inhibitors; ARBs: angiotensin receptor blockers

Variable		Diabetic Ulcer TN=32 n (%)	No Diabetic Ulcer TN=569 n (%)	P-value	Test Value
Age, median (IQR)		64.0 (58.25–68.5)	61.0 (54–68)	0.334**	8181
Sex	Male	22 (6.5)	318 (93.5)	0.153***	2.040
	Female	10 (3.8)	251 (96.2)		
Nationality	Bahraini	28 (5.5)	485 (94.5)	0.725****	0.124
	Non-Bahraini	4 (4.5)	84 (95.5)		
Smoking	Yes	9 (10.7)	75 (89.3)	0.021***	7.753
	No	23 (4.4)	494 (95.6)		
Alcohol	Yes	2 (15.4)	11 (84.6)	0.219***	3.034
	No	30 (5.1)	558 (94.9)		
Type of diabetes	1	0 (0)	20 (100)	0.281****	1.163
	2	32 (5.5)	549 (94.5)		
Duration in years, median (IQR)		15 (10–20)	11 (7–20)	0.184**	7795
Insulin	Yes	22 (9.8)	203 (90.2)	<0.001***	14.149
	No	10 (2.7)	366 (97.3)		
Metformin	Yes	18 (3.9)	448 (96.1)	0.003***	8.794
	No	14 (10.4)	121 (89.6)		
Glucagon-Like Peptide (GLP-1) agonists	Yes	2 (6.5)	29 (93.5)	0.502****	0.082
	No	30 (5.3)	540 (94.7)		
Sodium-Glucose Cotransporter-2 inhibitors	Yes	0 (0)	54 (100)	0.103****	3.337
	No	32 (5.9)	515 (94.1)		
Dipeptidyl Peptidase-4 (DPP-4) inhibitors	Yes	13 (5.1)	243 (94.9)	0.817***	0.054
	No	19 (5.5)	326 (94.5)		
Sulfonylurea	Yes	11 (4.2)	248 (95.8)	0.306***	1.048
	No	21 (6.1)	321 (93.9)		
Statins	Yes	26 (5.4)	455 (94.6)	0.860***	0.031
	No	6 (5)	114 (95)		
ACEI/ARBs	Yes	14 (5.3)	250 (94.7)	0.983***	<0.001
	No	18 (5.3)	319 (94.7)		
Aspirin	Yes	19 (10.3)	165 (89.7)	0.001***	13.160
	No	13 (3.1)	404 (96.9)		
Clopidogrel	Yes	0 (0)	7 (100)	0.680****	0.398
	No	32 (5.4)	562 (94.6)		
Calcium channel blockers	Yes	4 (3.2)	121 (96.8)	0.369***	1.413
	No	28 (5.9)	448 (94.1)		
Diuretic	Yes	4 (5.3)	71 (94.7)	0.583****	<0.001
	No	28 (5.3)	498 (94.7)		
Beta blockers	Yes	1 (2.3)	43 (97.7)	0.302****	0.877
	No	31 (5.6)	526 (94.4)		
Essential Hypertension	Yes	21 (5.2)	380 (94.8)	0.892***	0.018
	No	11 (5.5)	189 (94.5)		
Dyslipidemia	Yes	25 (5.4)	441 (94.6)	0.935***	0.007
	No	7 (5.2)	128 (94.8)		
Chronic Kidney Disease	Yes	3 (13.6)	19 (86.4)	0.077****	3.130
	No	29 (5)	550 (95)		
Ischemic Heart Disease	Yes	8 (13.1)	53 (86.9)	0.004***	8.173
	No	24 (4.4)	516 (95.6)		
Sensory symptoms*	Yes	8 (5.5)	137 (94.5)	0.835***	0.014
	No	24 (5.3)	432 (94.7)		
Intermittent claudication	Yes	0 (0)	36 (100)	0.248****	2.154
	No	32 (5.7)	533 (94.3)		
Rest pain	Yes	1 (11.1)	8 (88.9)	0.391****	0.607
	No	31 (5.2)	561 (94.8)		
Monofilament Test	Normal	24 (4.2)	549 (95.8)	<0.001***	31.485
	Abnormal	8 (28.6)	20 (71.4)		
Eye Exam—Retina screening	Normal	24 (4.7)	488 (95.3)	0.095***	2.783
	Abnormal	8 (9)	81 (91)		
Doppler Ultrasound	Normal	29 (4.9)	557 (95.1)	0.010****	6.573
	Abnormal	3 (20)	12 (80)		
Skin Abnormalities	Yes	18 (17.6)	84 (82.4)	<0.001***	37.005
	No	14 (2.8)	485 (97.2)		
Foot deformities	Yes	0 (0)	5 (100)	0.779****	135.614
	No	29 (4.9)	563 (95.1)		

As shown in Table 4, patients with foot ulcers had higher fasting blood glucose (p=0.047) and albumin-creatinine ratio levels (p=0.001), but lower HDL (p=0.009), eGFR (p=0.022), and hemoglobin levels (p<0.001).

**Table 4:** Association Between Laboratory Characteristics and Presence of Diabetic Foot Ulcers * Mann-Whitney test

Laboratory Test	Overall	Diabetic Foot Ulcer Present TN=32 n (%)	No Diabetic Foot Ulcer TN= 569 n (%)	P-value	U Test Value
Fasting Blood Glucose (mmol/L)	7.20 (6.0–9.40)	8.35 (6.55–11.73)	7.20 (5.95–9.10)	0.047*	7114
Hemoglobin A1C (mmol/mol)	57.00 (46–71)	65.00 (47–80.25)	57.00 (46–70)	0.055*	7182.5
Total Cholesterol (mmol/L)	4.00 (3.4–4.6)	3.95 (3.2–5.3)	4.00 (3.4–4.6)	0.691*	8616.5
Low-density lipoprotein (mmol/L)	2.09 (1.6–2.6)	2.09 (1.54–3.14)	2.09 (1.56–2.58)	0.748*	8658
High-density lipoprotein (mmol/L)	1.07 (0.9–1.3)	0.95 (0.77–1.25)	1.08 (0.93–1.28)	0.009*	6523
Triglycerides (mmol/L)	1.55 (1.1–2.1)	1.80 (1.03–2.48)	1.50 (1.1–2.1)	0.363*	8134.5
Vitamin B12 (pmol/L)	253.80 (208.3–326)	249.30 (217.03–327.5)	253.80 (207–326)	0.754*	8300.5
Albumin-Creatinine Ratio (mg/mmol)	1.10 (0.5–3.9)	8.45 (1.03–38.95)	1.00 (0.5–3.45)	0.001*	5138
Thyroid Stimulating Hormone (mIU/L)	1.94 (1.38–2.99)	1.89 (1.02–3.13)	1.96 (1.39–2.89)	0.995*	8659
Sodium (mmol/L)	139.00 (138–141)	140.00 (137.25–141)	140.00 (138–141)	0.847*	8779.5
eGFR (mL/min/1.73 m^2^)	94.00 (76–111)	79.00 (50–106.5)	94.00 (76.5–111)	0.022*	6772.5
Hemoglobin (g/dL)	13.20 (12.2–14.5)	12.15 (11.05–13.30)	13.20 (12.25–14.5)	<0.001*	5776.5

As shown in Table 5, the final multivariable logistic regression model indicated that insulin users were more likely to have diabetic foot ulcers than non-users (OR 2.555, p=0.042). Patients with ischemic heart disease (OR=3.752, P=0.016) and skin changes (OR=8.166, P<0.001) had a markedly increased risk of diabetic foot ulcers. Additionally, lower hemoglobin levels were independently associated with diabetic ulcer (OR=1.424, P=0.002).

**Table 5:** Predictors of Diabetic Foot Ulcer Among Patients With Diabetes Mellitus: Final Step of Logistic Regression Analysis * Inversely coded Variables entered in step 1: Smoking, Insulin, Metformin, Aspirin, Ischemic Heart Disease, Monofilament Test, Doppler Ultrasound (Arterial Flow)/Palpation, Skin Abnormalities, Fasting Blood Glucose (mmol/L), HDL (mmol/L), ACR (mg/mmol), Hemoglobin (g/dL), GFR.

Variable	Odds Ratio (95% Confidence Interval)	P-value
Smoking	1.638 (0.571–4.699)	0.359
Insulin	2.555 (1.033–6.319)	0.042
Metformin	2.223 (0.897–5.504)	0.084
Ischemic heart disease	3.752 (1.277–11.022)	0.016
High-density lipoprotein	3.552 (0.788–16.008)	0.099
Hemoglobin*	1.424 (1.143–1.774)	0.002
Skin changes	8.166 (3.407–19.572)	<0.001

## Discussion

This study aimed to determine the prevalence and predictors of foot ulcers among patients with diabetes attending PHCs in Bahrain. The results showed that approximately 5% of the participants had diabetic foot ulcers. In addition, higher rates of diabetic foot ulcers were noted among patients with foot skin changes, ischemic heart disease, low hemoglobin, and insulin users.

Compared with the present study, some previous studies reported higher rates of diabetic foot ulceration; however, these comparisons should be interpreted cautiously because study populations, definitions, and outcomes differed across reports [5,6,8]. In contrast, lower rates were also reported in the literature. For example, a study conducted in Kuwait, Qatar, and Saudi Arabia reported a diabetic foot prevalence of less than 3% [7]. The wide range of diabetic foot ulcer prevalence could be attributed to several factors, such as different populations, as some studies included only patients with type 2 DM, while others involved patients with type 1 DM as well. Additionally, differences in settings, patients’ characteristics, diagnostic criteria, and awareness about foot care practices may have also contributed to the discrepancies noted.

As expected, skin abnormalities were found to correlate with diabetic foot ulcers. Similarly, many studies found that skin changes, including tinea pedis, calluses, and skin dryness, were associated with diabetic foot ulcers [11-13]. According to ADA guidelines, inspection for these changes is essential, as they increase the risk of diabetic foot ulcers [10]. As expected, patients with ischemic heart disease had a higher risk of suffering from diabetic foot than their counterparts. This could be explained by the fact that ischemic heart disease and diabetic foot ulcers share the same risk factor profile and pathogenesis [14]. Similar findings were reported in the literature [15]. For example, a systematic review of 10 studies found a bidirectional association between ischemic heart disease and diabetic foot ulcers [16]. Interestingly, some studies have revealed that the more severe diabetic foot ulcers are, the higher the risk of cardiovascular disease [17].

In the present study, lower hemoglobin levels were independently associated with increased odds of diabetic foot ulcers. Similarly, a study of approximately 650 patients found that low hemoglobin levels were associated with diabetic ulcers [18]. In addition, some studies found that low hemoglobin was a risk factor for diabetic foot-related amputation [18,19]. Possible reasons for anemia in patients with diabetic foot ulcers include chronic inflammation, which impairs iron utilization and inhibits erythroid precursor proliferation, as well as other factors such as recurrent infections and repeated surgical debridement [18,19].

Here, insulin use was found to be linked to diabetic foot ulcer formation. A possible reason for this finding is that insulin is often prescribed for patients with long-standing or poorly controlled diabetes, who are more likely to suffer from neuropathy, peripheral vascular disease, and impaired wound healing. Similar findings were also reported in the literature [6-8].

Although univariate analysis suggested associations between smoking, diabetes duration, and diabetic foot ulcer, these relationships were not retained in the multivariable logistic regression model. However, several previous studies found significant relationships between smoking, diabetes duration, and the risk of diabetic foot ulcers. Surprisingly, the present study did not find any significant association between age and the risk of developing diabetic foot ulcers. In contrast, other studies reported such associations.

This study has several notable strengths. It included a relatively large sample drawn from patients with diabetes attending all PHCs in Bahrain. It assessed comprehensive clinical and laboratory data to identify relevant predictors of diabetic foot ulcers. For instance, neuropathy was assessed through symptoms and the monofilament test, while vascular function was evaluated using Doppler findings. In addition, it is one of the few studies to examine the prevalence of diabetic foot ulcers and their associated factors in Bahrain. However, this study has some limitations. Some variables, such as body mass index, foot care practices, socioeconomic status, and a history of ulcers, were not assessed. In addition, the cross-sectional nature of the study design limits the assessment of causality.

## Conclusions

In conclusion, the prevalence of diabetic foot ulcers among patients with diabetes attending PHCs in Bahrain was relatively low. Diabetic foot ulcers were more common among patients with skin changes, ischemic heart disease, insulin use, and lower hemoglobin levels. These findings highlight the importance of early detection of foot abnormalities and regular screening for high-risk patients in primary care settings. Routine screening, particularly for patients with high-risk profiles, should be conducted by a multidisciplinary diabetes care team to reduce morbidity and prevent severe outcomes. Further longitudinal and multicenter studies are needed to establish causal relationships and to explore additional risk factors associated with diabetic foot ulcers.
